# Hiding in plain sight: the F segment and other conserved features of seed plant SK_n_ dehydrins

**DOI:** 10.1007/s00425-017-2679-7

**Published:** 2017-03-20

**Authors:** G. Richard Strimbeck

**Affiliations:** Department of Biology, Norwegian University of Science and Technology, 7491 Trondheim, Norway

**Keywords:** LEA protein, Localization, Membrane binding, Phosphorylation, Sequence conservation

## Abstract

**An 11-residue amino acid sequence, DRGLFDFLGKK, is highly conserved in a subset of dehydrins found across the full spectrum of seed plants and here given the name F-segment.**

An 11-residue amino acid sequence, DRGLFDFLGKK, is highly conserved in identity and polarity in 130 non-redundant dehydrin sequences representing conifers and all major angiosperm groups. This newly described motif is here given the name F segment based on the pair of hydrophobic F residues at the core of the sequence. The majority of dehydrins previously classified as SK_n_ dehydrins contain one F segment N terminal to the S and K segments and can accordingly be reclassified as FSK_n_ dehydrins. A cysteine-containing variant, GCGMFDFLKK, occurs in a few rosid and asterid taxa. The S segment in this and other dehydrin types also includes previously overlooked conserved features, including a KLHR prefix and charged or G residues within and following the characteristic string of S residues. Secondary structure prediction models indicate that the F segment and S segment prefix may form amphipathic helices that could be involved in membrane or protein binding.

## Introduction

Dehydrins are a family of land plant proteins that may be expressed constitutively at low levels but are often produced de novo or at higher levels in response to drought, low temperature, or other stresses. They have been detected by western blotting or nucleic acid sequencing in all types of land plants, including bryophytes, pterophytes, gymnosperms, and all major groups of angiosperms. The main structural features of dehydrins were first described over 20 years ago (Close 1996), and include the number and modular arrangement of three types of short, distinctive segments in the protein, designated K, Y, and S segments. Using the YSK shorthand nomenclature proposed by Close, most known dehydrins fall into five types: K_n_, SK_n_, K_n_S, Y_n_K_n_, and Y_n_SK_n_,.

With a very few exceptions (noted below) the common feature of all dehydrins is one or more repeats of the K segment, a lysine-rich, 15 amino acid sequence with a highly conserved pattern of charged and nonpolar residues. The consensus K segment sequence in angiosperms is EKKDIMGKIKEKLPG, with a generally similar pattern conserved in conifer variants (Jarvis et al. 1996; Perdiguero et al. 2014). The identity or polarity of all K segment residues is highly conserved. The K segment forms an amphipathic α-helix in nonpolar environments and binds membranes (Koag et al. 2003, 2009; Eriksson et al. 2011), suggesting that a primary function of dehydrins is to protect membranes and perhaps proteins against dehydration stress.

The Y segment comprises seven residues with the consensus sequence (V/T)DEYGNP. When present, usually one to three closely spaced copies of the Y segment are located towards the N terminal end of the protein relative to the S or K segments. A group of four *Cornus sericea* dehydrins contain from 14 to 35 recognizable Y segments (Sarnighausen et al. 2004), and there are a few dehydrins with four.

Unlike the K and Y segments, as a rule the S segment occurs only once in any one dehydrin. It is recognized as a series of from three to nine consecutive serine residues, often interrupted by a single, usually polar residue, often D or E, after the first S. It is located N-terminal to the first K segment or at the C-terminus. Dehydrins with S segments have been found in all major seed plant taxa and the moss *Physcomitrella patens*. These segment types are bracketed and separated by unconserved regions, sometimes called phi segments. These can vary in length from a few to over 100 residues, and are rich in glycine and polar residues, so that dehydrins as a group are highly hydrophilic.

These basic features are found in the several hundred complete dehydrin sequences in online databases, and have been discussed and reaffirmed in numerous review articles on dehydrins (Close 1996; Allagulova et al. 2003; Rorat 2006; Eriksson and Harryson 2011). There are only a few known exceptions to the five dehydrin groups described by Close (1996), for example a K_4_SK_2_ dehydrin in *Picea abies* (GenBank accession ABS58630.1), an SK_3_S variant in *Stellaria longipes* (CAA79709.1), and a Y_2_KY_2_KY dehyrdrin in *Juglans regia* (AGJ94410.1). Also only few proteins with recognizable Y or S segments and sequence similarity to other dehydrins but with truncated or completely lacking K segments are described. These include a group of conifer proteins (Perdiguero et al. 2014), a Y_14_S protein in *C. sericea* (Sarnighausen et al. 2004), and complete ORF sequences with terminal S segments but no clearly identifiable K segments in the *Prunus persica* (XP 007202808) and *Solanum lycopersicum* (XP 010317810.1) genomes. Perdiguero et al. (2012) recently reported on two additional conserved segments in Pinaceae SK_n_ dehydrins.

While compiling a library of dehydrin amino acid sequences, I noticed a short, palindromic sequence in many SK_n_-type dehydrins: GLFDFLG, located near the N-terminal end of the protein. Further inspection suggested a longer conserved sequence with the preliminary 11 residue consensus DRGLFDFLGKK. Here I report that the majority of SK_n_ dehydrins contain a single copy of this segment, which I have named the F segment, and can thus be designated FSK_n_ dehydrins. I decribe its taxonomic breadth and sequence conservation and variation. I also note additional sequence conservation around the S segment in both FSK_n_ and Y_n_SK_n_ dehydrins.

## Materials and methods

To compile a library of dehydrin sequences, I searched the NCBI data for sequences identified as dehydrins and conducted BLAST searches on the angiosperm and conifer consensus K segment sequences. The results were screened to identify and remove sequences lacking core dehydrin characteristics, allelic variants, and highly similar homologs within taxa. Genera such as *Pinus*, *Vitis*, and *Solanum* are overrepresented in NCBI by registration of dozens of allelic variants and nearly identical homologous sequences in closely related taxa. Based on the number and occurrence of K, Y, and S segments, the resulting 395 sequences were classified into the five types described by Close, with the few exceptions noted. It was in this process that I first noticed the F segment.

After a BLAST search in the NCBI database for proteins containing variations of the 11-residue sequence DRGLFDFLGKK, I screened the results as above to remove non-dehydrins and redundant variants within taxa. Sequences were aligned using Clustal Omega and the alignments checked and adjusted manually. To explore sequence variation in and around the conserved segment, I isolated and aligned a 32-residue sequence centered on it and tallied the amino acids of each type found at each position.

To explore variation in and around the S segment, all S-segment containing dehydrins from the library were extracted. I excerpted a 35-residue segment centered on the consecutive S residues. These excerpts were manually aligned using the first S residue, recognizable variants of a commonly occurring HR prefix, and the first occurrence of a charged residue, usually D or E, following the S residues. Alignment of the latter was forced by inserting blanks in sequences with fewer than the maximum number of nine S residues. Following alignment, I tallied the amino acids at each position for each of the four dehydrin types (including FSK_n_) that incorporate S segments.

The eight-structure class protein secondary structure prediction model SSpro8 (Magnan and Baldi 2014) was run on a selection of full-length FSK_n_ dehydrin sequences to assess the potential for helical or sheet structures in and around the F and S segments.

## Results and discussion

### Frequency of the F-segment in dehydrins

In the initial screening and classification of 395 minimally redundant dehydrin sequences recovered from NCBI, 93 were classified as SK_n_ type dehydrins based on the presence of S and K but no Y segments (Table 1). I found F segments in 82 of these, indicating that most known SK_n_ type dehydrins can be reclassified as the FSK_n_ type. There were only nine FK_n_ type dehydrins in the sample, and only one with more than one copy of the F segment, an F_3_SK_2_ dehydrin in *Rhododendron* (AGI36547.1). There were no sequences with both Y and F segments. An additional 75 sequences that were incomplete at the N terminal end could potentially be any of a number of types including FK_n_ or FSK_n_ type dehydrins. About 25% of the 72 complete gymnosperm dehydrin sequences in the library contained F segments, but none contained Y segments (Table 1). The absence of Y segments in gymnosperms was noted by Perdiguero et al. (2012, 2014), but not in earlier reviews of dehydrin occurrence, structure, and function (e.g. Close 1996; Allagulova et al. 2003; Rorat 2006; Eriksson and Harryson 2011). There were also no complete gymnosperm SK_n_ or K_n_S dehydrins in the curated library, so that the available data indicates that in gymnosperms the S segment is always preceded by an F segment.

**Table 1:** Distribution of dehydrin types in a curated library of 395 minimally redundant dehydrins retrieved from NCBI

Dhn type	Angiosperm	Gymnosperm	Total	% of total
?K_n_ + ?SK_n_	37	38	75	19.0
K_n_	27	9	36	9.1
K_n_S	25	0	25	6.3
SK_n_	11	0	11	2.8
FK_n_	3	6	9	2.3
FSK_n_	65	17	82	20.8
Y_n_K_n_	37	0	37	9.4
Y_n_SK_n_	113	0	113	28.6
Uncommon types	5	2	7	1.8
Total	323	72	395	

“?” indicates incomplete N terminal sequences lacking F and Y segments

### Conservation variation in the F segment

The BLAST search of the NCBI database for proteins containing variations of the 11-residue sequence DRGLFDFLGKK yielded an initial library of about 575 complete and partial sequences, about 300 of which were readily recognized as dehydrins based on the presence of one or more recognizable variants of the K segment. After screening out redundant sequences, I arrived at a list of 130 minimally redundant proteins representing a broad spectrum of seed plant groups, including conifers as representative of gymnosperms and a variety of groups within the monocot, caryophyllid, rosid, and asterid clades of angiosperms. 123 of these also contained an S segment.

**Fig. 1:**
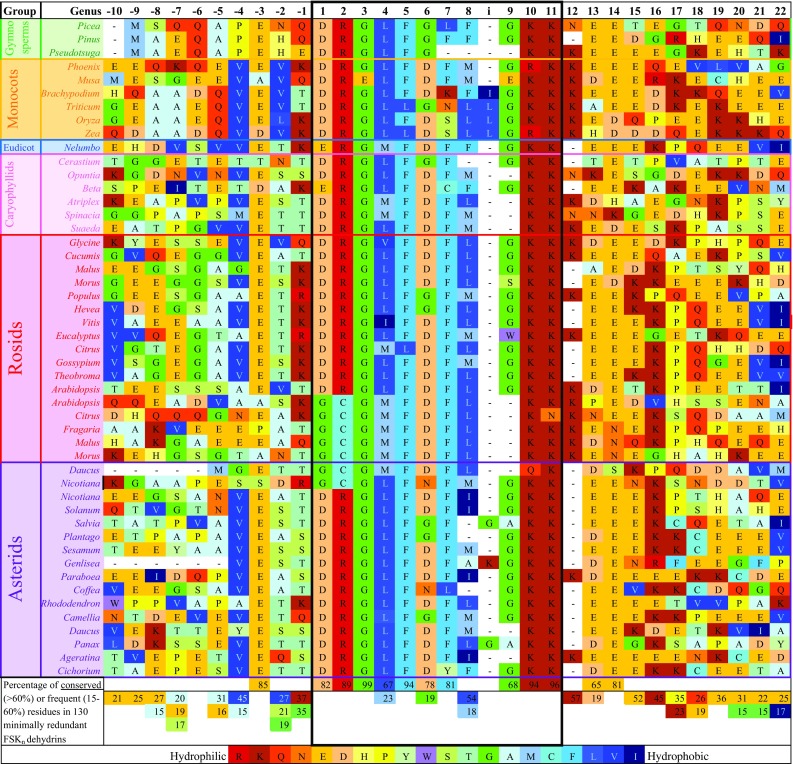
Alignment, conservation, and variation in 32-residue aligned sequence excerpts from selected seed plant taxa centered on the F segment in FSK_n_ dehydrins. The “i” in the numbered residue positions in the *top row* indicates a presumed insertion in Poaceae. Residues are ordered and colored by Kyte-Doolittle hydrophobicity (Lefranc 2017). The F segment is outlined in black

The 11-residue DRGLFDFLGKK sequence specified in the BLAST search is well-conserved across all 130 sequences, with the core 6-residue sequence GLFDFL nearly invariant in identity or polarity in all variants across the taxonomic spectrum (Fig. 1). The G following this core sequence is deleted in about 40% of the sequences, and there is an apparent insertion of a second hydrophobic residue after the conserved F/L in position 8 in Poaceae. In a subset of 12 rosid sequences and two asterid sequences, the initial DR is replaced by GC and the second G is omitted, giving the consensus GCGMFDFLKK in this variant. The conservation of a rare and metabolically expensive cysteine residue suggests some additional functionality associated with this couplet. The terminal K residues, typically followed by three to five more charged residues, can be interpreted as general features of dehydrins but may also contribute to the functionality of the segment. Similarly, a well-conserved E residue at position −3 may contribute some functionality.

The F segment seems to have gone unnoticed over the ca. 25 years of investigation into dehydrin structure and function. To give credit where it is due, Perdiguero et al. (2012) noted a 23-residue N-terminal sequence that includes the F segment and appeared to be conserved in conifers and, with considerable indel variation, in a few angiosperms. My analysis indicates that the core F segment is highly conserved in seed plants, and I propose that it should be recognized as a fourth functional segment type in the dehydrin family.

### Conserved features of the S segment

The S segment has been previously characterized as a string of three to as many as 13 serine residues, and typically followed by a string of charged K, D, or E residues (Eriksson and Harryson 2011). In K_n_S dehydrins it is located at the C-terminus of the protein. In other types it is N-terminal to the first K segment and in between it and F or Y segments where these are present, as indicated by nomenclature.

**Fig. 2:**
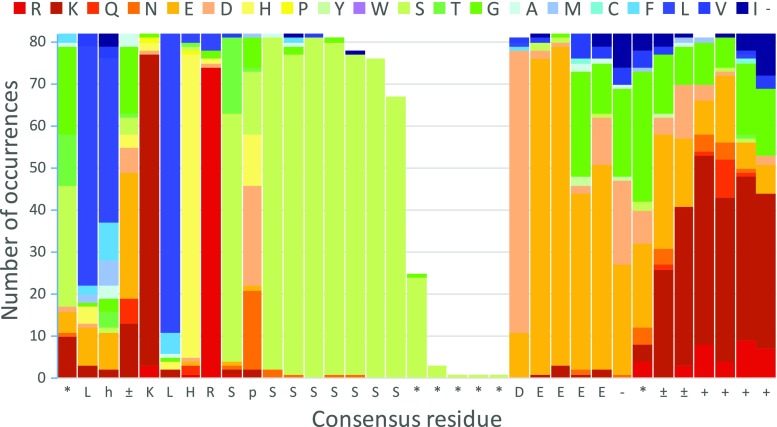
Sequence conservation and variation in S segments from 82 FSK_n_ dehydrins. *p* polar, *h* hydrophobic, *plus or minus sign* charged, *minus sign* acidic, *plus sign* basic, *asterisk* no consensus

Additional conserved features around the S segment emerge on closer examination. In dehydrins with S segments on the N terminal side, the first S in the sequence is typically followed by a D, E, or G residue, which is then followed by two to as many as 12 additional serine residues. In K_n_S dehydrins, this is mirrored by a highly conserved terminal DSD motif, which, in the 25 K_n_S sequences in my curated sample, always forms the C-terminus of the protein. In FSK_n_ dehydrins, there is a well-conserved KLHR prefix (Fig. 2), and the C terminal S is followed by 2–7 acidic residues, giving the generalized sequence KLHRS(D/E)S_2–12_ (D/E)_2–7_, followed by a string of predominantly G and charged residues. The prefix is somewhat less conserved in Y_n_SK_n_ and SK_n_ dehydrins, where the initial K is not conserved and a polar residue may replace H. K_n_S dehydrin S segments have a more variable mix of charged, H, and G residues as a prefix.

### Structural modeling

Predicted structures from SSpro8 (Magnan and Baldi 2014) for a selection of FSK_n_ dehydrins consistently suggest a short helical region centered on the five residue core sequence LFDFL in the consensus F segment and the GCG variant, including those where the D is replaced by a G residue. In a helical wheel projection of this short sequence, the four hydrophobic residues are arrayed on one side of the helix, with the charged D or somewhat hydrophilic G residue opposing them. This observation suggests that the F segment could have amphiphilic membrane or protein binding properties similar to those of the K segment. The same model also consistently predicts helical regions N terminal to the S segment. The conserved pattern of hydrophobic residues in this region (Fig. 2) could result in an amphiphilic structure here as well. All K segments are also consistently helical in the SSpro8 predictions.

### Conservation implies function

The high degree of conservation in the K, Y, F, and S segments in these otherwise highly variable and unstructured proteins implies that all four segment types play important roles in the overall function of the protein. Functional studies show that that the K segment binds membranes and therefore suggest that the main function of dehydrins is membrane protection (Koag et al. 2003, 2009). Membrane binding may be modulated by phosphorylation of S and pH-dependent dissociation of H residues (Eriksson et al. 2011), suggesting a function for the conserved H and S residues in the S segment.

The functions of the Y segment remain a mystery. Close et al. (1993) found some similarity between the Y segment and a nucleotide binding site in bacterial chaperones, an observation that has been echoed in some reviews (e.g. Allagulova et al. 2003; Rorat 2006). That similarity is not at all obvious on inspection of the sequences in the original report (Martin et al. 1993), and in any case there seems to have been no follow-up on this assertion. Whatever its function, gymnosperms are able to survive in a wide range of environments, including extremely dry or cold conditions, without any apparent need for the Y segment.

As noted above, the F segment may form a short, amphipathic helix with membrane or protein binding properties, and the S segment prefix may also have amphipathic properties. Sucrose, raffinose, and various compatible solutes have been hypothesized to stabilize (Carpenter and Crowe 1988) or replace (Clegg 1985) hydration shells around proteins or membranes; perhaps dehydrins play a similar role. Alternatively, binding by K segments or other amphipathic regions could anchor dehydrins to membranes or proteins so that they could act as “molecular spacers” (Strimbeck et al. 2015), preventing close approach and conformational or phase changes associated with repulsive forces under dehydration (Wolfe and Bryant 1999). Protein modification and binding studies (e.g. Koag et al. 2003, 2009; Eriksson et al. 2011) or other protein modification experiments may help clarify the binding and related protective properties of the F segment and other conserved regions of the different dehydrin types.

#### *Author contribution statement*

GRS conducted all of the original analyses reported in this article.
